# False-Negative Motor-Evoked Potential Due to Contrast-Induced Encephalopathy During Coil Embolization for Intracranial Aneurysm: A Case Report

**DOI:** 10.7759/cureus.73487

**Published:** 2024-11-11

**Authors:** Shigeki Takada, Takahiko Kamata, Haruki Yamashita, Shota Ogasawara, Nobutake Sadamasa, Waro Taki

**Affiliations:** 1 Department of Neurosurgery, Koseikai Takeda Hospital, Kyoto, JPN; 2 Department of Neurosurgery, Kyoto University Graduate School of Medicine, Kyoto, JPN; 3 Department of Clinical Laboratory, Koseikai Takeda Hospital, Kyoto, JPN

**Keywords:** cerebral aneurysm, coil embolization, contrast-induced encephalopathy, false-negative, motor-evoked potential

## Abstract

Contrast-induced encephalopathy (CIE) is a rare neurological complication that can occur following the use of contrast media during angiographic procedures. It can lead to neurological deficits, such as motor weakness. Transcranial motor-evoked potential (TcMEP) monitoring is commonly used to detect pyramidal tract disorders during embolization for intracranial aneurysms. However, it remains unclear whether TcMEP monitoring is effective in cases of motor weakness associated with CIE. We present a case of a false-negative motor-evoked potential caused by CIE during coil embolization for unruptured internal carotid artery aneurysms. A 68-year-old woman with dizziness underwent an MRI, which revealed multiple unruptured cerebral aneurysms, including a right anterior choroidal artery aneurysm (Ach AAn) and a posterior communicating artery aneurysm (Pcom AAn). Coil embolization was performed for the right Ach AAn and Pcom AAn with intraoperative TcMEP monitoring under general anesthesia. Throughout the procedure, no abnormalities were detected in the TcMEP monitoring. A total of 360 mL of contrast medium was used. After regaining consciousness, the patient experienced left hemiplegia and unilateral spatial neglect. Cerebral angiography revealed no missing branches, including those of the Ach A and Pcom A. MRI showed no acute ischemic or hemorrhagic changes. However, CT revealed CIE. The left hemiplegia and unilateral spatial neglect gradually improved and were completely resolved by postoperative day four. This case suggests that CIE-associated motor weakness may not be detected by intraoperative TcMEP monitoring. Therefore, in patients who develop motor weakness immediately after coil embolization for intracranial aneurysms without changes in TcMEP, brain CT should be performed to exclude CIE.

## Introduction

Contrast-induced encephalopathy (CIE) is a rare but well-recognized neurological complication associated with contrast medium exposure during various angiographic procedures. CIE can lead to neurological deficits, including motor weakness, sensory disturbances, seizures, unilateral spatial neglect, visual disturbances, aphasia, and unconsciousness. Generally, CIE is a reversible condition, and the associated neurological deficits resolve within a few days [1]. Motor-evoked potentials (MEPs) are commonly used to monitor motor function during procedures under general anesthesia. MEP monitoring includes both transcranial MEP (TcMEP) and transcortical MEP, with TcMEP being particularly useful for detecting ischemic complications related to pyramidal tract disorders during coil embolization for intracranial aneurysms [2,3]. However, it remains unclear whether TcMEP monitoring is effective in detecting motor weakness associated with CIE. We report the case of a patient who developed hemiplegia following coil embolization for unruptured internal carotid artery (ICA) aneurysms, despite intraoperative TcMEP monitoring.

## Case presentation

History and examination

**Figure 1:**
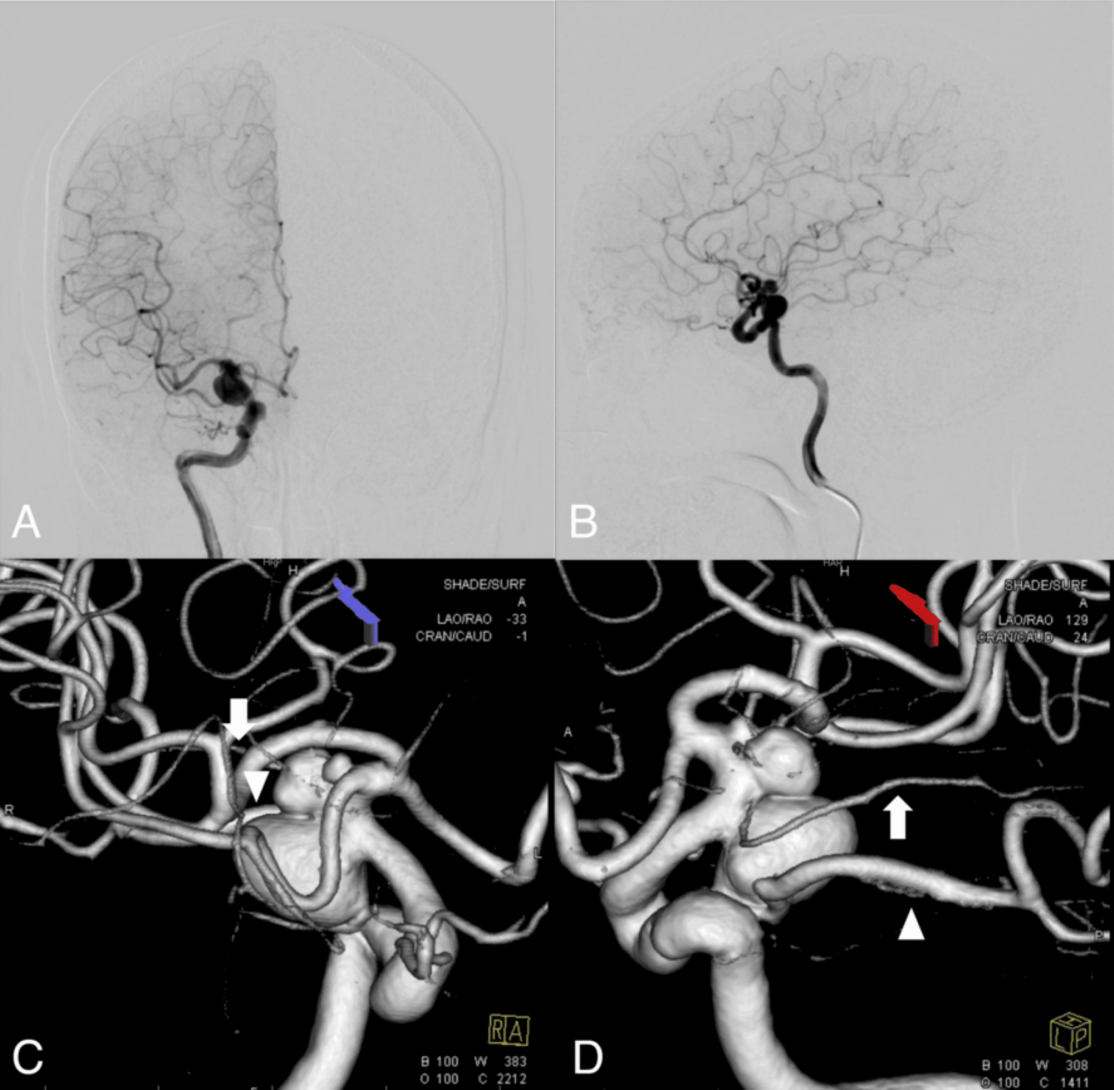
Multiple ICA aneurysms on the right side A right ICA angiogram revealed multiple aneurysms (A and B). Three right ICA aneurysms were identified on 3D rotational angiography, including an Ach AAn and a large Pcom AAn (C and D). White arrows and arrowheads indicate the Ach A and the posterior cerebral artery arising from the Pcom A. Ach Aan, anterior choroidal artery aneurysm; ICA, internal carotid artery; Pcom AAn, posterior communicating artery aneurysm

A 68-year-old woman presented with dizziness. MRI revealed multiple unruptured cerebral aneurysms. She was referred to our hospital for endovascular treatment. The patient had no medical history of hypertension, renal dysfunction, or stroke. A right ICA angiogram showed three right ICA aneurysms, including an anterior choroidal artery aneurysm (Ach AAn) (neck, 3.42 mm; height, 5.63 mm; and width, 5.99 mm) and a posterior communicating artery aneurysm (Pcom AAn) (neck, 4.93 mm; height, 8.96 mm; and width, 11.87 mm) (Figure 1A-1D). Based on clinical scales for aneurysm rupture prediction, the risk of rupture in these aneurysms was considered relatively high. Therefore, coil embolization of the Ach AAn and Pcom AAn with TcMEP monitoring under general anesthesia was planned.

TcMEP monitoring techniques

TcMEP was elicited via transcranial electrical stimulation of the motor cortex using corkscrew needle electrodes. A lateral C3-C4 montage, based on the international 10-20 system, was employed for stimulation. A high-voltage stimulator (SEN-4100, Nihon Kohden Corporation, Tokyo, Japan) was used to elicit multiple TcMEPs. To obtain MEP waves, a train of five constant-voltage 0.05-ms-wide stimuli, delivered at 2-ms interstimulus intervals, was used. The stimulus intensity was set at the beginning of the surgery and adjusted to a suprathreshold level for each stimulus. The myogenic response to TcMEP stimulation was recorded using patch electrodes placed on the abductor pollicis brevis muscle of both hands. The bandpass filter was set to 20-2,000 Hz. The baseline MEP was recorded after the induction of general anesthesia, and subsequent MEPs were recorded following placement of the guiding catheter and insertion of the microcatheter and coil.

Operation

Due to insurance calculation issues in Japan, single-staged flow-diverting stents with concurrent coil embolization for aneurysms are not accepted. Therefore, coil embolization followed by staged flow diversion was planned. Coil embolization was performed under general anesthesia, induced via intravenous infusion of propofol and remifentanil. The right femoral artery was accessed using a guiding sheath (6-Fr FUBUKI Dilator Kit, Asahi Intecc Co., Ltd., Nagoya, Japan), introduced over a hydrophilic 0.035-in soft-tip-angled guidewire, and navigated into the right ICA. A distal access catheter (6-Fr SOFIA SELECT 115 cm, MicroVention-Terumo, Tustin, California, USA) was introduced over the AXS Offset (Stryker, Kalamazoo, Michigan, USA), and a microguidewire (CHIKAI 14 200 cm, Asahi Intecc Co., Ltd.) was placed in the cavernous portion of the right ICA. A microballoon (SHOURYU HR 4 mm × 7 mm, Kaneka Medix Corporation, Osaka, Japan) was advanced into the right ICA using a microguidewire (TENROU S10-14 200 cm, Kaneka Medix Corporation). A microcatheter was then advanced into the AAn using a microguidewire (CHIKAI 14 200 cm, Asahi Intecc Co., Ltd.).

**Figure 2:**
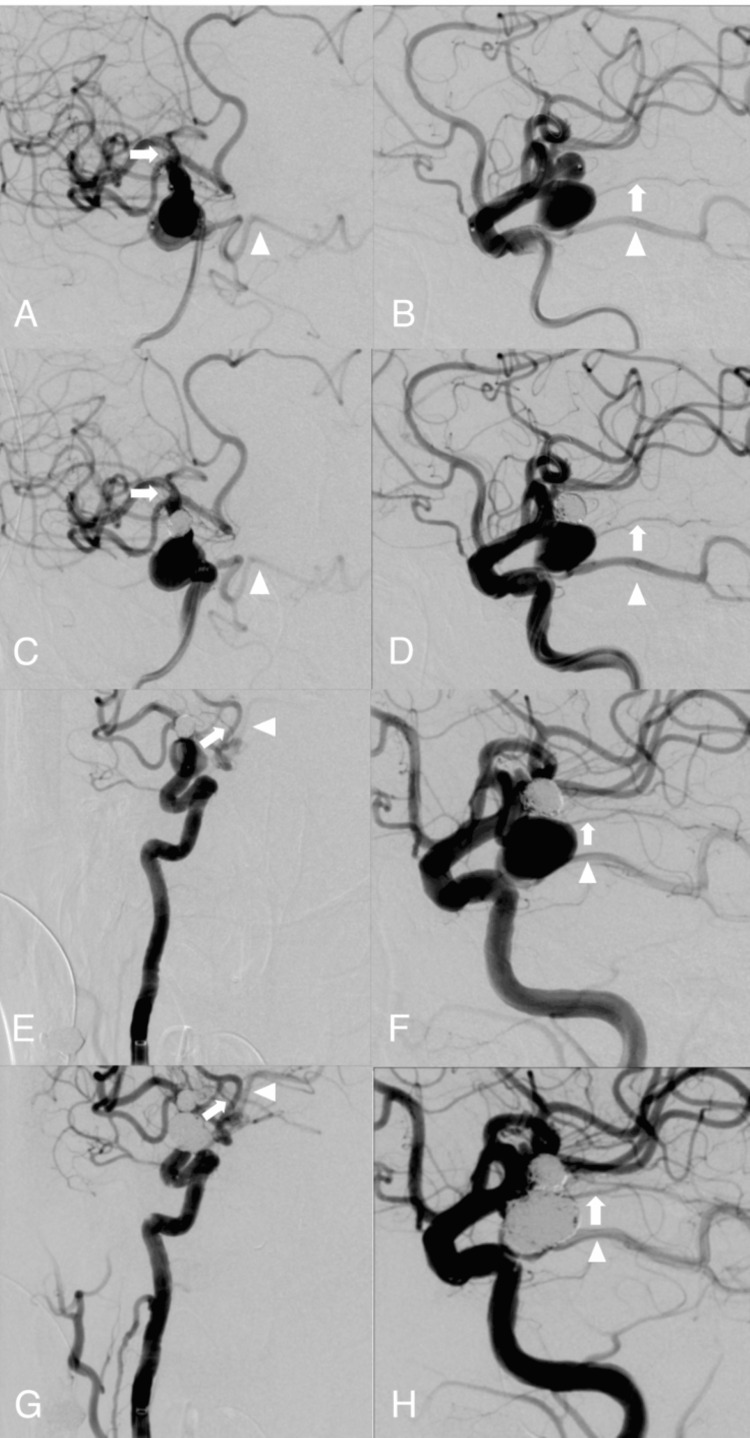
Intraoperative angiography of the right ICA Preoperative (A and B) and postoperative (C and D) angiograms during coil embolization of an Ach AAn are shown, with the missing branch not detected. Preoperative (E and F) and postoperative (G and H) angiograms during coil embolization of a Pcom AAn are also shown, with the missing branch not detected. White arrows and arrowheads indicate the Ach A and the posterior cerebral artery arising from the Pcom A. Ach Aan, anterior choroidal artery aneurysm; ICA, internal carotid artery; Pcom AAn, posterior communicating artery aneurysm

**Figure 3:**
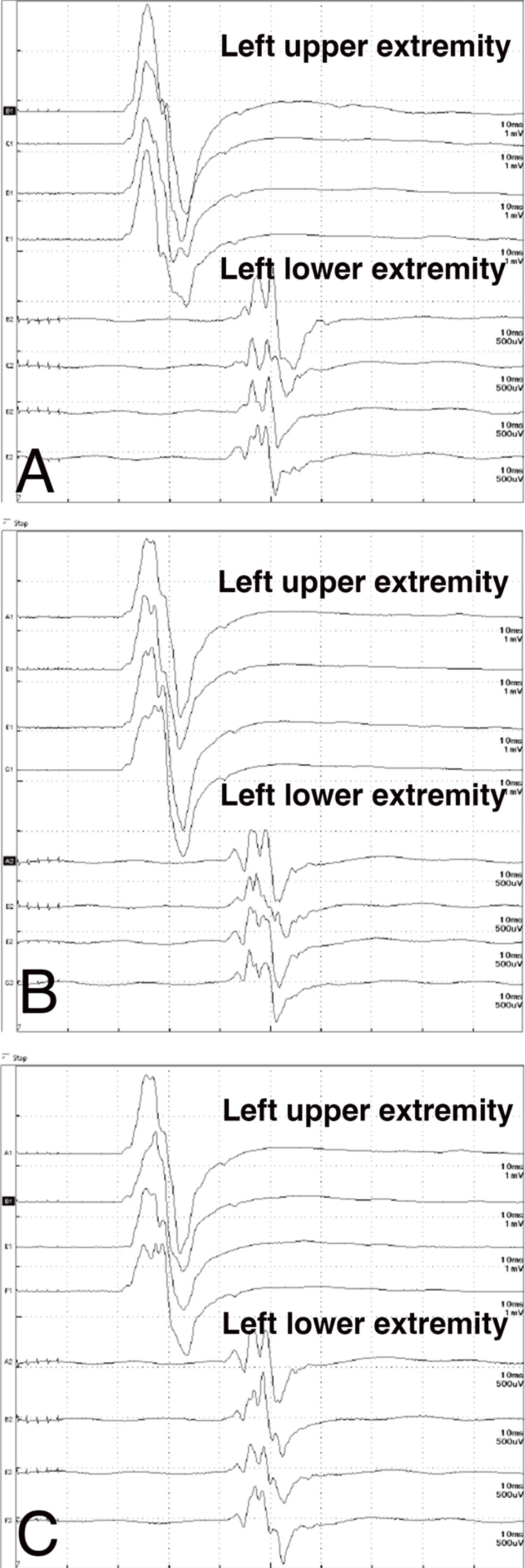
TcMEP monitoring throughout the procedure There was no difference in the MEP wave of the left upper and lower extremities before (A) and after (B) coil embolization of the Ach AAn. Similarly, the MEP wave remained unchanged following coil embolization of the Pcom AAn (C). Ach AAn, anterior choroidal artery aneurysm; MEP, motor-evoked potential; Pcom AAn, posterior communicating artery aneurysm; TcMEP, transcranial motor-evoked potential

First, coil embolization was performed for the Ach AAn using the balloon-assisted technique. Before detaching each coil, a contrast medium was injected and the MEP amplitude was checked. The Ach A was angiographically preserved, and the MEP wave remained unchanged throughout the procedure (Figure 2A-2D, Figure 3A, 3B). Second, coil embolization was performed for the Pcom AAn using the double catheter and stent-assisted technique. The left femoral artery was accessed using a guiding sheath (4-Fr FUBUKI Dilator Kit, Asahi Intecc Co., Ltd.), introduced over a hydrophilic 0.035-in soft-tip-angled guidewire, and navigated into the right vertebral artery (VA). A distal access catheter (TACTICS 125 cm, Technocrat Corporation, Hiroshima, Japan) was placed in the right VA. A microcatheter (Headway Duo 156 cm, MicroVention-Terumo) was navigated from the ipsilateral PCA into the middle cerebral artery via the Pcom A. Two microcatheters (Excelsior SL-10 STR 150 cm, Stryker; Headway 17 STR 150 cm, MicroVention-Terumo) were inserted into the Pcom AAn using a microguidewire (CHIKAI 14 200 cm, Asahi Intecc Co., Ltd.) through the 6-Fr SOFIA SELECT 115 cm (MicroVention-Terumo) in the right ICA. After coil embolization, a Neuroform Atlas 3 mm × 15 mm (Stryker) was deployed from the ICA to the Pcom A to stabilize the coil mass. A right ICA angiogram revealed that the Pcom A was patent, both aneurysms were satisfactorily occluded, and no branches were missing (Figure 2E-2H). Throughout the coil embolization, no reduction or disappearance of the MEP wave amplitude was observed (Figure 3A-3C). Hemostasis was achieved at the right femoral artery access site using Perclose (Abbott Laboratories, Chicago, Illinois, USA). A total of 360 mL of contrast medium (Iohexol, Omnipaque®) was used during the procedure.

Postoperative course

**Figure 4:**
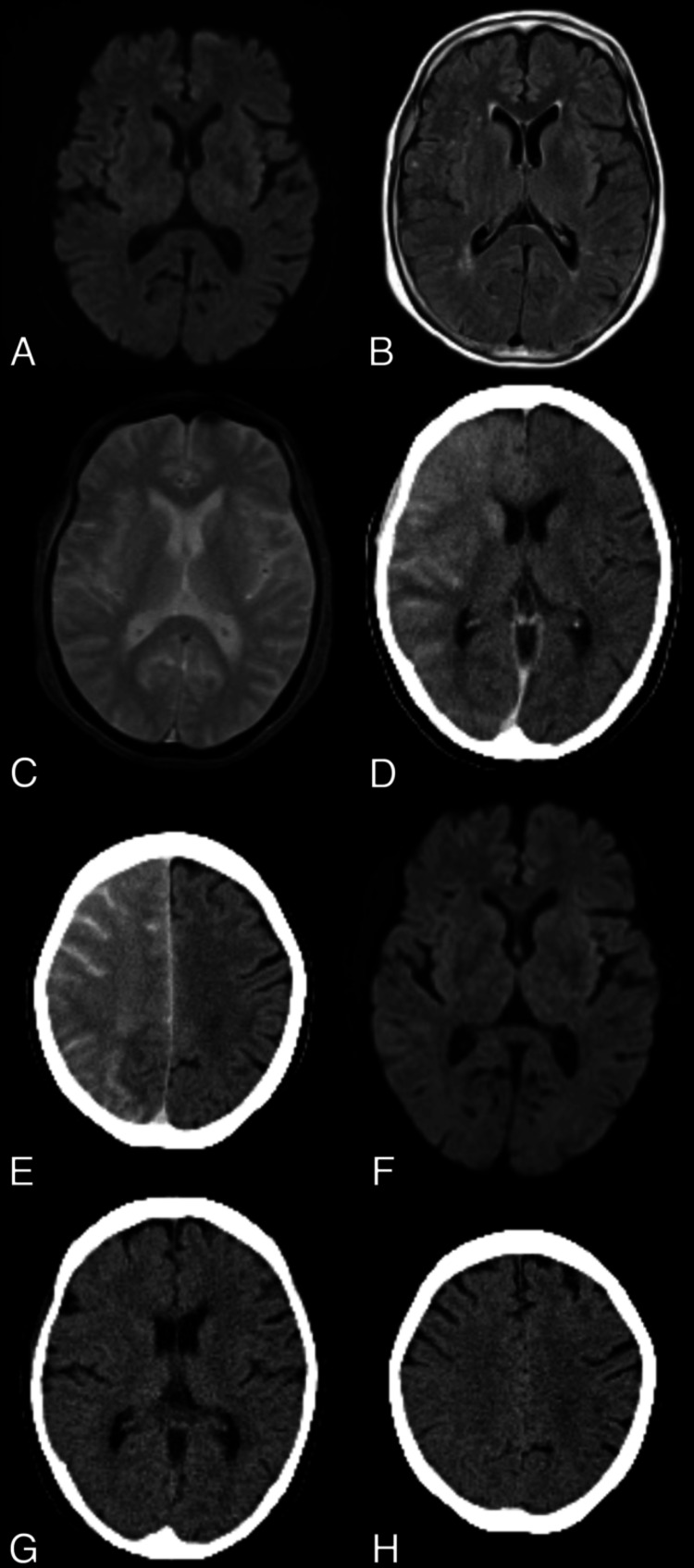
Postoperative MRI and CT findings DWI (A), fluid-attenuated inversion recovery (B), and T2*-weighted imaging (C) revealed no acute ischemic or hemorrhagic changes. Noncontrast CT, performed within one hour after coil embolization, showed cortical and subcortical contrast enhancement in the right cerebral hemisphere (D and E). DWI on postoperative day one (F) showed no ischemic changes. The contrast enhancement in the right cerebral hemisphere on CT completely resolved by postoperative day four (G and H). DWI, diffusion-weighted imaging

After extubation, the patient developed left hemiplegia and unilateral spatial neglect. Cerebral angiography revealed no missing branches, including the Ach A and Pcom A. Additionally, MRI showed no acute ischemic or hemorrhagic changes (Figure 4A-4C). Noncontrast CT, performed within one hour after coil embolization, revealed cortical and subcortical contrast enhancement in the right cerebral hemisphere (Figure 4D, 4E). The patient was diagnosed with cerebral ischemia and edema and was managed with supportive care. Diffusion-weighted imaging on postoperative day 1 showed no ischemic changes (Figure 4F). Over the following days, the left hemiplegia and unilateral spatial neglect gradually improved. The contrast enhancement in the right cerebral hemisphere on CT and the neurological deficit completely resolved by postoperative day four (Figure 4G, 4H). The patient was discharged home without any neurological deficits on postoperative day seven.

## Discussion

This is the first report of CIE following coil embolization for cerebral aneurysms that presents with a false-negative intraoperative TcMEP result. The underlying mechanisms and causes of CIE remain unclear. Previous studies suggest that CIE may be associated with transient blood-brain barrier breakdown and increased permeability, which could allow contrast medium to extravasate into the central nervous system, leading to cerebral edema and altered neuronal excitability [4-8]. Risk factors for CIE have not been widely established. However, prior studies have identified hypertension, renal dysfunction, and a history of stroke as potential risk factors for CIE [9-11]. Kim et al. proposed that large aneurysms, longer procedure times, and higher contrast doses are also risk factors for CIE [12]. The present case did not include a history of hypertension, renal dysfunction, or stroke, but the internal carotid-posterior communicating artery aneurysm was large (>10 mm), the procedure time was long (321 minutes), and the contrast dose was 360 mL.

In this case, CT performed immediately after coil embolization for right ICA aneurysms revealed cortical, subcortical, and basal ganglia contrast enhancement in the right cerebral hemisphere, suggesting that these regions were affected by the contrast medium. The patient exhibited left hemiplegia and unilateral spatial neglect, symptoms consistent with CIE. TcMEP monitoring is commonly used to detect pyramidal tract disorders during coil embolization for intracranial aneurysms. However, in this case, motor weakness was not detected by TcMEP monitoring, resulting in a false-negative outcome. Two mechanisms could explain this false-negative result. First, direct stimulation of deeper structures within the subcortical motor pathways may bypass the affected lesion, leading to a false-negative result. If the lesion affected by the contrast medium is bypassed during stimulation, TcMEP monitoring may fail to detect motor weakness. Second, motor weakness could result from lesions outside the pyramidal tract, including the premotor and supplementary motor areas (extrapyramidal pathways). The pyramidal tract descends through the corona radiata, internal capsule, cerebral peduncles, basis points, and medullary pyramids to the spinal cord, and damage to these regions significantly alters MEP, often resulting in hemiplegia. A previous study reported that ischemic lesions in the premotor area could cause motor deficits without significant changes in MEP. Motor weakness from infarction in the supplementary motor areas and pathways may be difficult to distinguish from that caused by direct involvement of the pyramidal tract [13]. In the present case, the premotor and supplementary motor areas were affected by the contrast medium, as evidenced by the brain CT obtained immediately after coil embolization. Therefore, these affected areas may have contributed to the contralateral motor weakness observed, suggesting an extrapyramidal disorder.

## Conclusions

We present a patient with CIE who developed hemiplegia following coil embolization for unruptured intracranial aneurysms, with intraoperative TcMEP monitoring. In this case, TcMEP did not detect CIE-associated motor weakness. This suggests that CIE-related motor weakness may not be identifiable through intraoperative TcMEP monitoring. If patients exhibit motor weakness immediately after coil embolization for intracranial aneurysms without changes in TcMEP, a brain CT should be performed to exclude CIE.
